# Comment on ‘The expression landscape of cachexia‐inducing factors in human cancers’ by Freire *et al*.

**DOI:** 10.1002/jcsm.12670

**Published:** 2021-01-13

**Authors:** Longxiang Xie, Xiangqian Guo

**Affiliations:** ^1^ Institute of Biomedical Informatics, Cell Signal Transduction Laboratory, Bioinformatics Center, Henan Provincial Engineering Center for Tumor Molecular Medicine, School of Basic Medical Sciences Henan University Kaifeng 475004 China

## Ethical Guidelines

All authors certify that they comply with the ethical guidelines for authorship and publishing in the *Journal of Cachexia, Sarcopenia, and Muscle*.^8^

We have read with great interest the recent excellent article by Freire and colleagues, which comprehensive analysed the gene expression profiling of cachexia‐inducing factors (CIFs) in 12 human cancers.^1^ The author concluded that differential expressed secretome genes may be potential mediators of cachexia, and the distinct gene expression landscape of CIFs can predict the unfavourable prognosis. Although we appreciate the authors' comprehensive data analysis, which is a worthwhile research, several issues about the methodologies used in their analysis should be noted, which may influence the soundness of their conclusion.

Firstly, the authors limited their study to The Cancer Genome Atlas (TCGA) and Genotype‐Tissue Expression databases (GTEx) and did not investigate other international public databases with mass expression profiling, including Gene Expression Omnibus (GEO), International Cancer Genome Consortium (ICGC), and ArrayExpress. Selection of a restricted subset of databases for characterizing the expression profile of secretome genes can lead to biased results, even wrong conclusions. Meanwhile, authors just concentrated on the mRNA profiling rather than protein expression levels of cachectic soluble factors. It will be better to analyse the protein expression levels of these genes based on reverse‐phase protein arrays (RPPAs) from The Cancer Proteome Atlas (TCPA)^2^ or ELISA experiment in plasma.

Secondly, the authors explored the expression profile of these 25 CIFs based on tumour purity caculated by ABSOLUTE^3^ and stated that the expression profile of CIFs is associated with tumour purity in PAAD. Although ABSOLUTE is an effective statistical method for predicting the tumour purity, it was developed based on SNP (single nucleotide polymorphism) array data.^3^ Nowadays, a number of statistical methods and software tools including ESTIMATE,^4^ AbsCN‐seq,^5^ and InfiniumPurify^6^ have been developed using gene expression, copy number variation, or DNA methylation data. Therefore, if the tumour purity of 12 cancers inferred by multiple methods/tools for multi‐omics level can be taken into consideration, a more reliable conclusion will be obtained.

Finally, the authors found that the altered expression of these 25 CIFs can predict the poor overall survival of 12 cancer types using the univariate Cox analysis. We do not think the evidence is clear enough, because this analysis was performed without considering any other clinical characteristics. It is better to perform univariate Cox analysis for clinicopathological factors (such as gender, age, clinical stages, or chemoradiotherapy) and then incorporate the prognostic factors identified by univariate Cox analysis (*P* < 0.1) using a stepwise selection/backward elimination process for multivariate Cox analysis.^7^ The multivariate Cox analysis results will help authors reveal whether abnormal 25 CIFs' expression is an independent predictors of cancer survival outcomes.

In conclusion, the authors analysed a valuable question regarding the CIFs in human cancers, but the results of this study should be explained with caution due to the limitations mentioned above.
